# Season of death, pathogen persistence and wildlife behaviour alter number of anthrax secondary infections from environmental reservoirs

**DOI:** 10.1098/rspb.2023.2568

**Published:** 2024-02-07

**Authors:** Amélie C. Dolfi, Kyrre Kausrud, Kristyna Rysava, Celeste Champagne, Yen-Hua Huang, Zoe R. Barandongo, Wendy C. Turner

**Affiliations:** ^1^ Wisconsin Cooperative Wildlife Research Unit, Department of Forest and Wildlife Ecology, University of Wisconsin-Madison, Madison, WI 53706, USA; ^2^ Norwegian Veterinary Institute, Ås, Norway; ^3^ College of Veterinary Medicine, University of Minnesota, Saint Paul, MN 55108, USA; ^4^ Department of Ecology and Evolutionary Biology, Yale University, New Haven, CT 06511, USA; ^5^ Institute for Biospheric Studies, Yale University, New Haven, CT 06511, USA; ^6^ US Geological Survey, Wisconsin Cooperative Wildlife Research Unit, Department of Forest and Wildlife Ecology, University of Wisconsin-Madison, Madison, WI 53706, USA

**Keywords:** *Bacillus anthracis*, disease transmission, environmentally transmitted pathogen, host–pathogen contact, reproduction number

## Abstract

An important part of infectious disease management is predicting factors that influence disease outbreaks, such as *R*, the number of secondary infections arising from an infected individual. Estimating *R* is particularly challenging for environmentally transmitted pathogens given time lags between cases and subsequent infections. Here, we calculated *R* for *Bacillus anthracis* infections arising from anthrax carcass sites in Etosha National Park, Namibia. Combining host behavioural data, pathogen concentrations and simulation models, we show that *R* is spatially and temporally variable, driven by spore concentrations at death, host visitation rates and early preference for foraging at infectious sites. While spores were detected up to a decade after death, most secondary infections occurred within 2 years. Transmission simulations under scenarios combining site infectiousness and host exposure risk under different environmental conditions led to dramatically different outbreak dynamics, from pathogen extinction (*R* < 1) to explosive outbreaks (*R* > 10). These transmission heterogeneities may explain variation in anthrax outbreak dynamics observed globally, and more generally, the critical importance of environmental variation underlying host–pathogen interactions. Notably, our approach allowed us to estimate the lethal dose of a highly virulent pathogen non-invasively from observational studies and epidemiological data, useful when experiments on wildlife are undesirable or impractical.

## 1. Introduction

Environmentally transmitted pathogens (ETPs) represent a large proportion of the most burdensome infectious disease agents globally [1]. Understanding their epidemiology often proves very challenging as they are difficult to detect, and for some, the environmental reservoirs are still poorly known [2,3]. Persistence in the environment is highly dependent on the traits of the pathogen and the characteristics of the environment. *Brucella abortus* bacteria can persist 20–80 days [4], *Toxoplasma gondii* oocytes can survive for months [5], and the prion responsible for scrapie can survive for at least 16 years [6]. Persistent pathogens in the environment can extend existing outbreaks or initiate ‘new’ outbreaks years into the future. For example, avian influenza outbreaks emerge in North America every 2–4 years, with emergence sparked by ingestion of virions from environmental reservoirs established in previous outbreaks [7,8]. Here, we estimate the number of secondary infections arising from pathogen reservoirs for a highly persistent ETP and how environmental variation affecting pathogen survival and host behaviours alters host–pathogen contact rates and, ultimately, transmission.

For directly transmitted pathogens, the basic reproduction number *R*_0_ is an estimate of the average number of secondary cases produced from a single infectious individual introduced into a susceptible population and is often used as a key epidemiological parameter [9]. However, the assumption of a naive population is not met for diseases in endemic areas, and estimating *R*_0_ for ETPs is arduous due to the extended infectious time in the environment, variation in the spatial extent of pathogen reservoirs, and heterogeneity in host movement, behaviour and susceptibility [10,11]. In cases where ETPs are only released into the environment at host death (i.e. obligate killer pathogens [12]), one host mortality will form one infectious reservoir ‘patch’ in the environment. We can assess the reproduction number *R*, which does not assume a naive population, defined here for ETPs as the average number of secondary infections produced by one infectious patch over its infectious period. This novel formulation of the reproduction number acknowledges that the population may not be entirely susceptible, without needing to know the susceptible proportion of the population to calculate the effective reproduction number *R_e_*. For persistent ETPs, patches can remain infectious spanning multiple, seemingly independent, outbreak events and monitoring of pathogen reservoirs is thus needed to identify how pathogen concentrations affect transmission risk over time [13].

In addition to pathogen persistence, information on host movement and behaviour is essential to identify contacts with pathogen reservoirs in heterogeneous landscapes that vary in exposure risk. Movement ecology studies of host locations can be combined with pathogen location data to estimate host–pathogen contact rates [14,15]. However, the temporal scales of host telemetry studies are often too coarse to determine an encounter with infectious patches present at a fine spatial extent. Fine-scale behavioural information like direct observation is needed to identify contacts with infectious patches in heterogeneous environments. For terrestrial vertebrates, the infectious sites often represent a small part of the host range, such as a water source or specific pasture sites [16,17], depending on how and where pathogens are released from hosts. Thus, reservoir-focused sampling techniques to monitor host behaviours and transmission risk can fill a gap in our understanding of transmission for ETPs.

Hosts may modulate their behaviour based on cues suggesting the presence of infectious patches. Exposure may be reduced if hosts avoid detectable cues associated with infection risk such as faeces, carcasses or macroparasites [18], a response now called the ‘landscape of disgust’ [19,20]. Conversely, exposure risk can be enhanced by attraction toward contaminated water or nutrient-rich foraging sites, leading to ingestion of pathogens with forage or water [16,21]. Because behaviour can depend on the state of the reservoir site, quantifying host–environment contact is crucial to assess disease transmission potential at heterogeneous environmental reservoirs.

*Bacillus anthracis* is a bacterial pathogen with two life forms; infectious spores that are maintained in the environment, and vegetative cells that multiply and cause disease inside mammalian hosts [22]. Spores at carcass sites can be found on grasses for several years [21] and in exposed surface soils for up to a decade [23]. A new case occurs when a host ingests spores while grazing at a carcass site [21,24], if the exposure exceeds the lethal dose threshold [25,26]. If exposed to a lethal dose, the host will die within a few days, and the site of death becomes a new infectious patch in the soil (an area less than 20 m^2^ [21]). For *B. anthracis*, estimating *R* is made conceptually tractable with this ETP being an obligate killer, where one host fatality generates one infectious patch that can then cause secondary infections. We aimed to estimate *R* for *B. anthracis*, incorporating variation in the three sides of the epidemiological triangle: host, pathogen and environment. We assessed host individual and population-level attraction to infectious sites using camera traps at anthrax carcass sites and paired controls sites, and quantified foraging behaviour of two host species, plains zebra (*Equus quagga*, hereafter zebra) and blue wildebeest (*Connochaetes taurinus*, hereafter wildebeest). We then estimated pathogen concentrations on soil and grasses at reservoir sites over a decade using interpolation and extrapolation from empirical datasets on soil and grass spore concentrations. Finally, we developed a simulation model to determine the exposure risk of the two host species to *B. anthracis* by estimating *R*, defined as the number of individuals a single anthrax infectious site may infect over its decadal lifespan. Knowing *R* and its duration for ETPs may inform wildlife disease management decisions, such as assessing spatial overlap with appropriate time lags at the interface between livestock and wildlife.

## 2. Material and methods

### (a) Study area

Etosha National Park (hereafter Etosha) is a 22 270 km^2^ nature reserve in northern Namibia containing a large salt pan surrounded by grasslands, shrublands and woodlands [27]. Etosha encompasses a sub-tropical, semi-arid savannah biome with a single wet season (January–April) and a long dry season (cool dry season, May–August and hot dry season, September–December). In Etosha, anthrax occurs mainly during wet seasons, through ingestion of spores by grazing herbivores [28–30]. The two main host species are plains zebra and blue wildebeest which represent 50.3%, and 16.2% of all confirmed anthrax cases recorded 1968–2020, respectively (2150 anthrax cases among all hosts; Etosha Ecological Institute; all data from this institute were communicated by Claudine Cloete in 2023). The population density of zebras is more than six times higher than wildebeests (estimate of 16 174 zebras and 2482 wildebeests [21]), and population estimates of both species have been fairly stable over the last 30 years [27]. Additional information about heterogeneities in anthrax dynamics within this study area is available [23,24,31].

### (b) Camera site data collection

Motion-sensing remote camera traps were used to collect animal behavioural data at carcass sites (study design and camera placement described in [21]). In brief, 13 rock-delimited 2.5 m radius zones at anthrax carcass sites (from 12 zebras and one wildebeest) paired with 13 control sites situated 100 m away were monitored between March 2010 and March 2013, totalling 14 779 days of observation (electronic supplementary material, figure S1). The sites were situated within the same habitat, thus, we assumed that visitation would not differ between the carcass and control sites. Motion-triggered cameras took 10 pictures at 1 s intervals continuously when movement was detected. Vegetation greenness for each site pair was obtained from the normalized difference vegetation index (NDVI) at a resolution of 250 m^2^ every 8 days (Global Inventory Modeling and Mapping Studies [32]).

Site visitation was recorded from photographs when animals entered the rock-delimited zones. The information recorded included date, time, species, age (juvenile: less than 1-year-old; sub-adult: 1–2 years old; adult: greater than 2 years old), sex, total time spent on site and time spent grazing in seconds. Age, sex, location in the image and coat patterns (electronic supplementary material, SI.1) were used to ensure whether the same individual was assessed across triggers proximate in time, defined as a gap of a few minutes. Depending on the position of the animal and the lighting of the image, age and sex could not always be determined, so the confidence of age/sex observations was ranked from 0 (impossible to identify) to 3 (certain), with confidence scores of 0 and 1 removed from relevant analyses. Two observers manually coded host demographics from images (C.C. and A.C.D.).

### (c) Host parameter analysis

We assessed the attractiveness of carcass sites to zebra and wildebeest from camera trap data, defining three behavioural response variables for both species, for each site: (i) monthly average number of visitations, (ii) monthly average probability of grazing given visitation and (iii) time spent grazing given grazing occurred (electronic supplementary material, SI.2). We investigated how the treatment (carcass or control), the number of months after death (MAD), environmental factors (NDVI and season), demographic variables (age and sex), and spatial variables (distance to the closest perennial water and distance to an edge of the main Etosha salt pan; electronic supplementary material, table S1) affected each of the three behavioural response variables, using generalized linear models. All analyses and models were conducted using the R software [33], with packages lme4 [34], and glmmTMB [35]. Only months with at least 20 days of recording for both cameras at a site (carcass and control) were included in the analysis. Out of the 546 individual camera-months sampled, 438 met this inclusion criterion. For included months with missing data, these had an average of 2.5 days missing (electronic supplementary material, SI.2, figure S2). The monthly visitation was analysed using a zero-truncated negative-binomial model, the proportion of monthly grazing events was analysed using a binomial model, and the time spent grazing using a log-transformed linear mixed model. Interactions between season and distance to water, as well as between treatment and MAD were included. In each model, we kept treatment, age and sex as fixed effects and site ID as a random factor. Model selection was made using the Akaike Information Criterion. For each response variable, 16 models were tested (electronic supplementary material, table S2), totalling 48 models per species.

### (d) Pathogen parameter estimation

Pathogen concentrations on grasses and soil, measured as colony-forming units (CFUs) per gram of dry matter, were obtained at zebra anthrax carcass sites (sampling method and data in [21] and [23]). Concentrations of spores on grasses were recorded at 23 sites up to 4 years after death. Two measurements were obtained from grasses, the concentration of pathogen on the aboveground material (i.e. everything above the roots), or in the upper portion of the aboveground material (the aboveground component after removal of 1 cm at the base of the plant which can collect soil; this upper grass portion is hereafter referred to as the ‘grass’ component ingested in simulations). Soil spore concentrations were recorded for up to 12 years at 40 carcass sites, but no spores were detected after 10 years [23].

To obtain estimates of spore concentrations on grasses and in soil over the full time series, we interpolated the soil CFU for all missing values (156 out of 442) at the 40 sites using the log-linear model from Barandongo *et al.* [23], with site age as a predictor. Then, we extrapolated the concentration of spores on grasses for all 40 sites over 10 years using two steps. We first fit a log-linear model using the soil CFU and age of sites to obtain the concentration of spores on the aboveground grass components:

2.1 $$\log_{10}({\mathrm{CFU}}_{\mathrm{aboveground},z,t}+1)=\;\log_{10}({\mathrm{CFU}}_{\mathrm{soil},z,t}+1)+\beta \times {\mathrm{Age}}_{z}+\;\varepsilon_{z,t},$$

where *β* is the regression coefficient, *ε* is the error at site *z* and time after death *t*. Then, we extrapolated the expected values of CFU on the grass tops, using a log-linear model based on the aboveground CFU only.

### (e) Reproduction number estimation: infection risk simulation model

**Figure 1.**
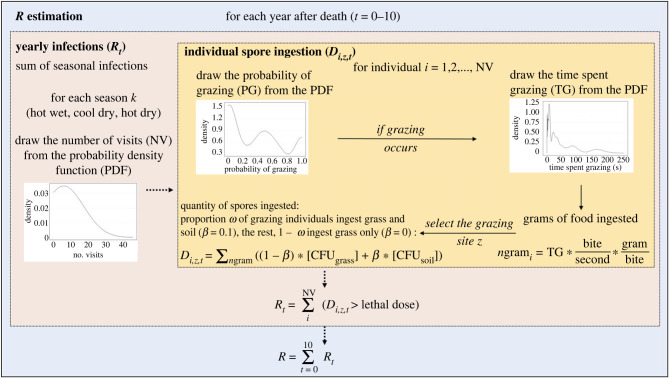
Model visualization for the estimation of the reproduction number *R* of anthrax in Etosha National Park, Namibia. Each simulation ran over 10 years and was repeated 100 times. Each year is separated into three seasons (*k*), the hot wet, cool dry and hot dry seasons. Each probability density function (PDF) is obtained using the camera trap data. During the hot wet season, the proportion of animals ingesting grass and soil *ω* is *ω*_wet_, while it is *ω*_dry_ during the cool and hot dry seasons. The yearly reproduction number *R_t_* is the sum of the number of infections occurring during each of the three seasons *k* of year *t*. The reproduction number *R* is the sum of the yearly reproduction number.

We estimated the reproduction number, *R*, as the number of lethal infections occurring at an infectious site over a decade by simulating host exposure to the pathogen. To simulate host behaviour, we drew the number of animals visiting an infectious patch, the probability of grazing, and grazing time from empirical probability density functions (PDFs). Then, to simulate individual host–pathogen exposure we estimated the quantity of spores ingested based on time spent grazing and seasonal patterns of soil ingestion [30]. We only considered an exposure to be an infection if the number of spores ingested, at one visitation, exceeded a fixed lethal dose; we did not consider multiple exposures. The estimated *B. anthracis* lethal dose by ingestion is between 10^5^ and 10^8^ spores [26], so we considered four threshold doses (10^5^, 10^6^, 10^7^ and 10^8^) to estimate which threshold matched the anthrax dynamic observed. Each model iteration simulated each season of each post-death year 0–10. A visual representation of the model is presented in figure 1. We ran each simulation 100 times to account for stochasticity.

The host empirical PDFs were built using the 3-year period of camera trap data and were subdivided using the significant variables obtained from the statistical analyses, mainly season, year after death and distance to water or salt pan (see main text result; electronic supplementary material, SI.3, figures S3–S8). Using a subdivided dataset allowed us to consider how host visitation and behaviours vary under a range of environmental conditions. We used PDFs rather than model predictions as we wanted to draw values from a distribution which encompasses the extreme values present in the data, better representing the stochasticity present in the system. From the statistical analysis, carcass sites influenced host visitations and grazing during the first 24 months, after which carcass and control sites did not differ. We assumed that behaviours at control sites, and at older carcass sites (i.e. greater than 2 years old, after the attraction signal has faded), were plausible estimates for visitation rates and behaviours at older carcass sites (i.e. 2–10 years in the simulation). To account for this temporal signal in host behaviour, we separated the datasets into three time periods: year 0, year 1 and years 2–10. To estimate host parameters for years 0 and 1, we used data from carcass sites for years 0 and 1 after death, respectively. For host parameters in years 2–10, we pooled data from control sites and for carcass sites over 2 years old.

Individual pathogen exposure was simulated by estimating the amount of forage ingested, then calculating the associated spore intake using empirical data. The number of grams ingested, *n*gram*_i_*, by individual *i*, is

2.2 $$n{\mathrm{gram}}_{i}=T_{i}\times Bn_{s}\times Bw_{s},$$

where *T_i_* is the time spent grazing by individual *i*, *Bn_s_* is the number of bites per second and *Bw_s_* is the weight of a bite for species *s*. Foraging zebras take bites of about two grams and an average of 27 bites min^−1^ [36,37] while wildebeest take bites averaging 1 g bite^−1^ and 26 bites min^−1^ [38,39]. To estimate the number of spores ingested by each individual, we randomly selected one of the 40 carcass sites for which we have spore concentrations as the site of foraging. Because zebra and wildebeest ingest not just grass, but also soil and roots when grazing [40,41], and because spore concentrations are higher in soil/roots than on grasses [21], we modelled the ingested spore concentration as a mix of grass and soil ingestion. The quantity of soil ingested while grazing at infectious patches is unknown, however, we expect hosts primarily consume grass when grazing. We assume that if soil was ingested, it would represent 10% of intake mass [26]. To denote this, we included a parameter *β* representing the portion of soil ingested per bite (*β* = 0.1 if soil ingestion occurs, *β* = 0 otherwise). For each gram ingested, we treated the concentration of spores in the grass and in the soil as a Poisson process (to counterbalance errors that arise from extrapolation without accounting for variance in this step, as in [26]), and we assumed that the concentration of pathogen at the site is not modified by grazing. Thus, we calculate the number of spores ingested, *D_i,z,t_*, by individual *i*, at infectious site *z*, of age *t* as

2.3 $$D_{i,z,t}=\;\overset{n{\mathrm{gram}}_{i}}{\underset{n=1}{\sum }}(\left(1-\beta )\;\mathrm{Pois}[{\mathrm{CFU}}_{\mathrm{grass},z,t}]+\;\beta \;\mathrm{Pois}[{\mathrm{CFU}}_{\mathrm{soil},z,t}\right]),$$

where *n*gram_i_ is the number of grams of food ingested, *β* is the proportion of soil ingested and CFU*_z,t_* is the number of spores ingested from grass and soil components. Because herbivores ingest soil more often during the wet season than the dry season [30], we included a weight *ω*_wet_ and *ω*_dry_ representing seasonal differences in the proportion of individuals that ingest soil while grazing, while others ingest grass only. We tested all combinations with *ω*_wet_ and *ω*_dry_ varying from 0 to 1, by 0.1 increments. For example, a simulation with the proportion *ω*_wet_ = 0.4 and *ω*_dry_ = 0 would simulate that 40% of grazing individuals ingest grass and soil while 60% ingest grass only during the wet season, and all grazing individuals ingest only grass during the dry season. Based on host foraging ecology [29,30], we assumed that the most likely parameter space for Etosha is within 0–40% of individuals ingesting soil when grazing, with a higher percentage during the wet versus dry season.

To further explore the role of environmental variability on *R*, we repeated simulations considering seasonality in the timing of reservoir formation and interannual variation in host foraging behaviour. The concentration of spores in soil reservoirs is over an order of magnitude lower when individuals die in dry seasons versus wet seasons [23]. Thus, to understand how the season of death impacts secondary infections, we separated infectious sites formed in dry seasons (five sites) from infectious sites formed in wet seasons (35 sites). Similarly, to explore interannual variation in *R*, we simulated the wet season under drought conditions using dry season behavioural data and under average rainfall conditions using wet season behavioural data. The camera trap data were collected during average to above-average rainfall years, however, in Etosha, zebras are less at risk of anthrax during drought due to changing habitat selection reducing exposure risk [31]. Under drought conditions, ‘wet season’ habitat use mirrors habitat use observed in dry seasons. With these changes, we considered five different data combination: (i) all data from hosts and infectious sites; (ii) all data from hosts and wet season formed infectious sites, (iii) all data from hosts and dry season formed infectious sites, (iv) data from hot dry host behaviour and wet season formed infectious sites and (v) data from hot dry host behaviour and dry season formed infectious sites.

Finally, to investigate which lethal dose threshold best-represented anthrax dynamics in the system, we simulated the number of cases that would be produced over 100 years, using infection model results combining zebra and wildebeest. We initialized the simulation using zebra and wildebeest anthrax mortality data from 2003 to 2013, representing 235 cases (Etosha Ecological Institute). For each time step, we obtained the number of yearly new cases by drawing the number of new infections occurring at each of the 235 sites from the infection model result, discriminating the sites by age. A maximum of 10 000 new infections per year was fixed, as it represents the average population of zebra and wildebeest present in Etosha (Etosha Ecological Institute). We used the model combination using all host and infectious sites to simulate this prediction. This is a simple model, used to inform where the lethal dose threshold might fall in free-ranging wildlife populations.

## 3. Results

### (a) Host parameters

**Figure 2.**
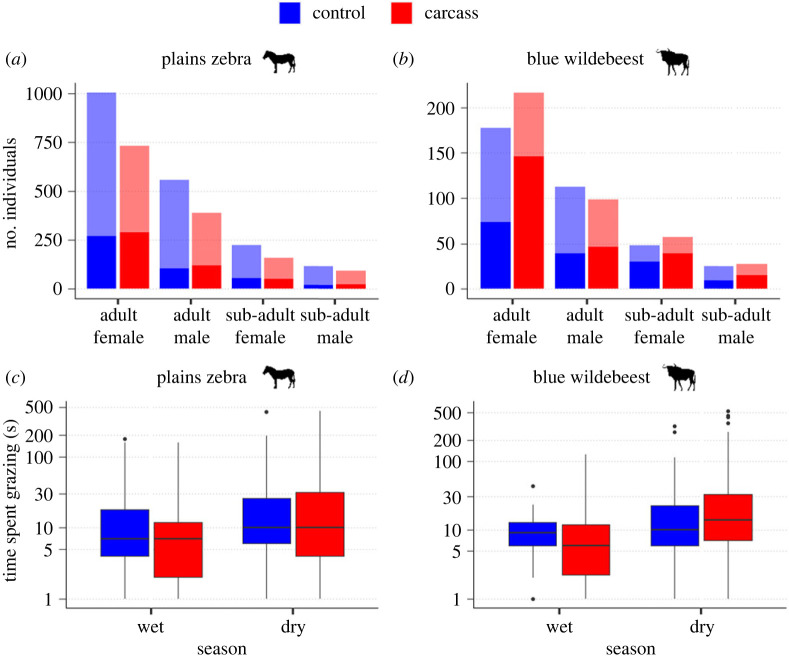
Total number of individuals by age and sex recorded visiting (lighter colours) and grazing (darker colours) at anthrax carcass sites (*a*,*b*), and the time spent grazing (in seconds) by season, at control (blue) and carcass (red) sites (*c*,*d*), for plains zebra (*Equus quagga: a*,*c*) and blue wildebeest (*Connochaetes taurinus: b*,*d*) in Etosha National Park, Namibia.

Of 119 226 triggers of the camera traps, 5196 contained zebras and 1043 contained wildebeests. Based on sequential triggers clustered in time, we identified visitations by 3838 zebras and 830 wildebeests. At visits to carcass sites, 37.2% of zebras grazed and 60.3% of wildebeests grazed, while at visits to control sites only 24.6% of zebras and 42.0% of wildebeests grazed. For both species, most recorded individuals were adult females (figure 2*a*,*b*; all statistical results in electronic supplementary material, SI.4). Among zebras, more individuals were recorded on sites closer to permanent water (*p* < 0.05), during the hot dry season (*p* < 0.0001) and at control sites (*p* < 0.05, figure 2*a*). For wildebeests, sites nearer the salt pan (*p* < 0.01) as well as carcass sites (*p* = 0.01) had more visitations (figure 2*b*).

Despite small visitation differences at carcass and control sites, the probability of grazing was significantly higher at carcass sites for both species (*p* < 0.0001; figure 2*a*,*b*). In addition, for zebra, the probability of grazing was higher for females (*p* < 0.0001), adults (*p* < 0.05), during the cool dry season (*p* < 0.05) and under higher NDVI (*p* < 0.0001). The probability of grazing at carcass sites significantly decreased the older the site (*p* < 0.001). Wildebeest grazing was similarly affected by NDVI (*p* < 0.05), and sites farther from permanent water significantly increased the probability of grazing during the dry season (*p* < 0.05).

Finally, given that grazing occurred, the time spent grazing for both species did not differ between site treatments. However, season played a major role in foraging time. Zebra grazed for longer times during both dry seasons (*p* < 0.001) than the wet season, while wildebeest spent longer times grazing during the cool dry season (*p* < 0.0001; figure 2*c*,*d*).

### (b) Pathogen estimation

**Figure 3.**
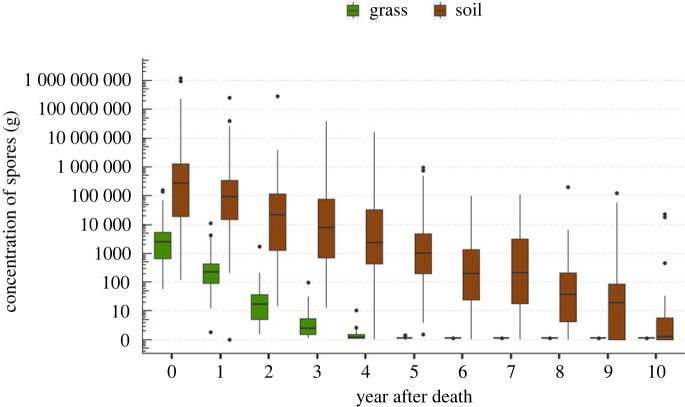
Decline in *Bacillus anthracis* spore concentrations at 40 anthrax carcass sites in Etosha National Park, Namibia, over time, on grass tops (green) and in surface soils (brown). Grass concentrations were obtained from extrapolation of the soil concentrations (equation 2.1).

For the 40 monitored carcass sites, we obtained a yearly concentration of spores per gram of soil or grass. For the soil pathogen concentrations, 35% of the data came from the model interpolation, while all the data presented for the grass tops came from the extrapolation. The concentration of spores on grasses was much lower than in soil, starting nearly three orders of magnitude lower (figure 3). Our data suggest that after 5 years, spores are absent from the grass, despite persisting in the soil.

### (c) *R* estimation

**Figure 4.**
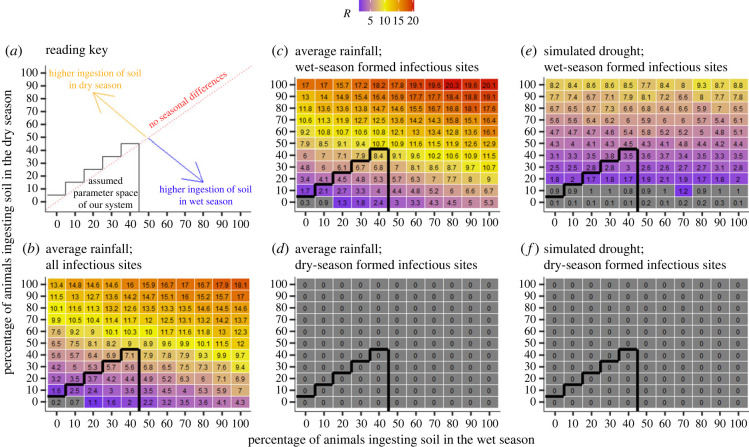
Variation in the reproduction number, *R*, for plains zebras (*Equus quagga*) at anthrax carcass sites, for a lethal dose threshold of 10^7^ *Bacillus anthracis* spores based on differences in seasonality of host visitation, season of reservoir formation, and percentage of individuals ingesting soil during grazing, estimated over the 10-year lifetime of a *B. anthracis* reservoir site. The percentage of animals ingesting soil while grazing is varied from 0% to 100%. In all cases, animals are ingesting 10% soil, 90% grass (by weight) if soil is consumed. Soil contact during foraging is considered for two seasons, wet season (numbers below the diagonal) and dry season (numbers above the diagonal). The *R* values falling along the diagonal represent no difference in soil exposure by season. The black line represents the most likely parameter space for the study system, according to host foraging literature [29,30]. The grey tiles represent an *R* < 1, then a colour gradient is used for *R* > 1. (*a*) reading key; (*b*) all host and reservoir data; (*c*) visitation rates under good rainfall, wet season reservoirs; (*d*) visitation rates under good rainfall, dry season reservoirs; (*e*) visitation rates under simulated drought, wet season reservoirs; (*f*) visitation rates under simulated drought, dry season reservoirs.

**Figure 5.**
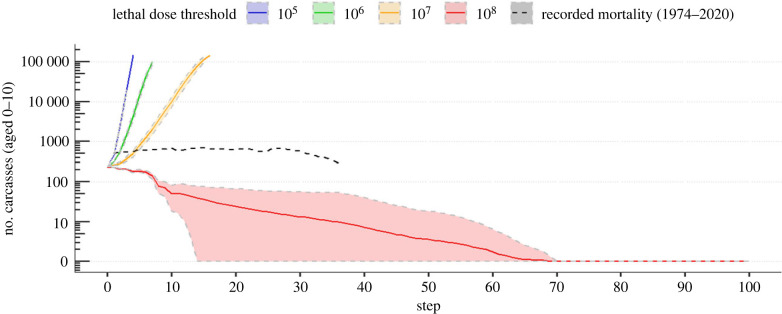
Prediction of the number of anthrax cases recorded over 100 years depending on lethal dose thresholds of 10^5^, 10^6^, 10^7^ and 10^8^. The shaded area represents the standard deviation over the 100 simulations. The black dashed line represents the recorded mortality in Etosha National Park, Namibia from 1974 to 2020. Each simulation started with the recorded mortality data from a decade (2003–2013). The *x*-axis represents the total number of potentially infectious carcass sites present in the environment (each site ‘disappears’ after 10 years). Predictions were made using the simulation model output, with a limit of 10 000 new cases. The lethal doses 10^5^, 10^6^ and 10^7^ lead to an explosion of cases, while the lethal dose of 10^8^ leads to extinction. Lethal doses maintaining a relatively stable number of cases over time would be between 10^7^ and 10^8^. The *y-*axis is log-transformed.

Across models, zebra had more anthrax infections than wildebeest, matching patterns observed in disease surveillance data. Since otherwise both species showed similar trends, we only show zebra results, and report wildebeest in electronic supplementary material. There was variability in *R* estimation due to the high heterogeneity in visitations, the proportion of individuals ingesting soil while grazing, and the lethal dose threshold considered (figure 4; electronic supplementary material, figures S9–S11). From our simulations, the lethal dose threshold most likely to match the dynamics observed in Etosha is between 10^7^ and 10^8^ spores for both species. Indeed, for a lethal dose threshold of 10^5^, 10^6^ and 10^7^, the estimated *R*, for our parameter space, is above two and we predict an exponential increase in anthrax cases over time, while, for a lethal dose of 10^8^, *R* is below one and we simulated pathogen extinction occurring after 70 years, on average (figure 5). If the lethal dose falls between 10^7^ and 10^8^ spores, extrapolating from our models, a host would need to ingest between 687 and 6878 g of grass only, or between 1.5 and 14.8 g of grass with soil (when soil is 10% of intake biomass) to become infected at a newly infectious site. The former is unlikely to occur based on site size and host behaviour, emphasizing the critical importance of soil ingestion for transmission risk.

Seasonality played a pivotal role in anthrax epidemiology and infectious site heterogeneity. In general, a larger proportion of individuals ingesting soil led to more infections, but the magnitude of this effect varied seasonally. For the same proportion of individuals ingesting soil during the dry or the wet season, more infections occurred during the dry season, possibly due to seasonal behavioural differences, with longer grazing times recorded during the dry season (compare *R* above versus below the diagonals, figure 4*b*; electronic supplementary material, figure S11b). Infectious sites formed in the wet season drove disease dynamics in this system (figure 4*c*,*e*; electronic supplementary material, figure S11c,e); and dry season formed infectious patches contributed no cases (all *R* = 0, figure 4*d*,*f*; electronic supplementary material, figure S11d,f), probably due to the lower spore concentrations found at dry season formed sites. Similarly, seasonality in host visitation rates played an important role in the number of individuals infected, with fewer infections during drought conditions (figure 4*e*; electronic supplementary material, figure S11e) than average rainfall conditions (figure 4*c*; electronic supplementary material, figure S11c), probably due to fewer visits and grazing events.

**Figure 6.**
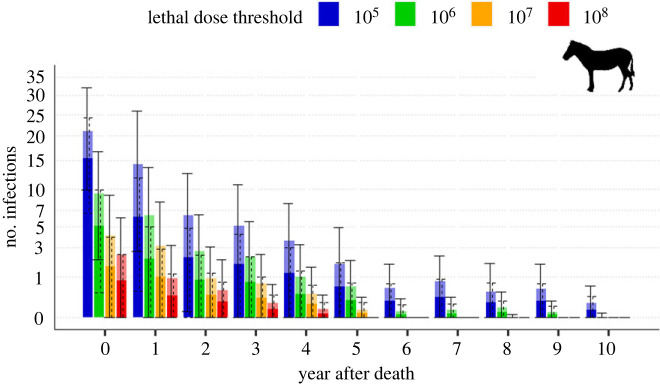
Variation in the average number of plains zebra (*Equus quagga*) secondary anthrax mortalities occurring per year after death at an anthrax carcass site, depending on the lethal dose threshold of *Bacillus anthracis* spores ingested. Opaque colours and dotted error bars represent the average number of infections within the assumed parameter space of our study system, i.e. the percentage of animals ingesting soil while grazing varied from 0% to 40%. The transparent bars and solid error bars represent the average number of secondary infections for the entire parameter space, i.e. the percentage of animals ingesting soil while grazing varied from 0% to 100%. The *y*-axis is square root transformed to better visualize small numbers. Error bars represent standard deviation.

The risk of infection diminished rapidly with site age (figure 6; electronic supplementary material, figure S12). If individuals only consumed grass, no infections were predicted after 3 years regardless of the lethal dose threshold or host species (electronic supplementary material, figures S13 and S14). However, when at least some individuals ingested soil when grazing, infections continued for longer. The higher the proportion of individuals ingesting soil, the higher the number of infections, occurring over a longer time since death. For a lethal dose threshold of 10^5^, infections occurred throughout the 10 years of simulation, while for thresholds of 10^6^, 10^7^ and 10^8^, no infections were recorded after years 9, 5 and 4, respectively.

## 4. Discussion

Determining fine-scale transmission dynamics for ETPs can be complex due to the difficulties in characterizing contact locations, reservoir infectiousness and frequency of interactions in heterogeneous environments. This study used simulation models combining long-term monitoring data of a pathogen in its environmental reservoir with fine-scale host behavioural data to estimate the number of secondary infections arising from infectious patches of an ETP. Together with knowledge of the long-term epidemiology of this system, this method has allowed us to evaluate variation in the number of secondary infections, *R*, arising from heterogeneities along the three sides of the epidemiological triangle (host, pathogen and environment). Our results suggest ecological mechanisms for why anthrax disease dynamics and seasonality can vary so dramatically in different ecosystems and offers a perspective on how ETP heterogeneities alter outbreak dynamics.

Investigating host individual behaviours at infectious reservoir sites revealed how fine-scale behavioural variation scales up to altered disease dynamics. Both host species had similar visitation rates between site treatments (at times even higher for zebra at control sites) and the time spent grazing did not differ between site treatments by age or sex. The critical difference in behaviour was that, over the study period, a higher proportion of animals chose to graze when encountering a carcass site: zebra by 1.5 times and wildebeest by 1.4 times. Notably, individuals were most likely to graze at infectious sites within 2 years after creation, when higher pathogen concentrations are more likely to result in a fatal exposure. This pattern goes against what would be expected under the landscape of disgust theory [19], as we see an increase in risky behaviour at infectious sites. Carcasses are known to enhance the nutrient content and palatability of the grasses at the site of death [42,43], and *B. anthracis* spores may further promote plant growth [44], increasing the chances of spores being ingested by a potential host. However, when focusing on the seasonality of anthrax infections, visitations and grazing probabilities were often higher during dry seasons than the wet season, suggesting a higher risk of infection during the dry seasons, contrary to what we observe in the system. The key difference in risk between seasons thus arises from soil contact, since herbivores ingest significantly more soil in wet than dry seasons [30]. *Bacillus anthracis* being a soil-borne pathogen, the quantity of soil a host ingests when grazing is a major component of transmission risk. Thus, one limit of our model comes from uncertainty in our estimate of the amount of soil ingested during foraging at an infectious patch (*β* parameter of equation (2.3)). While the quantity will vary depending on host and environmental factors, our estimate for this parameter was based on the faecal analysis from Turner *et al*. [30] and does not account for seasonal differences in forage digestibility. However, by varying the proportion of individuals ingesting soil, we explore the effect of spore exposure from soil in addition to spore exposure from grasses on *R*, and how this varies in different seasons. A finer assessment of the proportion of individuals ingesting soil at reservoir patches, the quantity of soil ingested, and the impact of seasonality on those variables across ecosystems would be important to increase our understanding of the disease transmission risk.

At the host population level, these behavioural patterns demonstrate two important findings. First, high variation in visitation rates suggests that encounters with infectious patches occur somewhat randomly at a local scale. Forage choice at the smallest spatial and temporal scales is an important driver of pathogen exposure; however, larger-scale factors such as proximity to desirable landscape features and seasonal changes in habitat selection affect the number of individuals available in an area to encounter infectious sites. Second, the expected *R* varies based on environmental conditions, which led to dramatically different disease dynamics in simulation models. Environment affects site infectiousness and host encounters, as well as spatio-temporal patterns in when and where host mortality occurs. Infectious sites created during the wet season have higher spore concentrations than those formed in the dry season [23], and in Etosha, dry seasons and drought shift hosts out of the high-risk area into habitat with lower anthrax risk ([31,45]; but see patterns in Tanzania, [46]). Infectious patches formed during the dry season did not contribute any new cases and host behaviours under drought-simulated conditions (based on dry season behaviours) significantly decreased *R* compared to average to wetter conditions, patterns that match disease outbreak dynamics in this ecosystem [24,47]. Thus, environmental variation in both infection potential of reservoir patches and host behaviour reinforces the wet season timing of anthrax in this ecosystem, where mortalities occurring in preferred wet season habitats are more likely to contribute secondary infections than mortalities occurring in dry season habitats.

The seasonality of anthrax outbreaks across the global range of *B. anthracis* varies from outbreaks associated with wet seasons or high rainfall events, to large, intermittent outbreaks often associated with dry seasons or droughts [48]. Our simulation results suggest underlying mechanisms that could drive variation in seasonality of outbreaks across ecosystems. For example, soil ingestion occurring during the dry season led to bigger outbreaks than during the wet season. This matches patterns observed across locations; outbreaks occurring during dry seasons are more epidemic-like, compared to the outbreaks occurring in the wet season that are typically smaller and more endemic-like [24,47]. This pattern could be explained by a seasonal behavioural change, with higher visitation rates during the end of the dry season increasing the exposed population, combined with longer times spent grazing, increasing individual exposure risk. We could then expect that extreme weather events such as drought could impact the disease dynamics in different ways. For environments where anthrax deaths peak during the dry season, drought would accelerate exposures and outbreak sizes, while for environments where anthrax death peaks during the wet season, drought would lead to a lower infection risk.

The intensity and frequency of anthrax outbreaks likewise vary across the global range of *B. anthracis*. In areas like Etosha where regular, small outbreaks maintain endemicity, contacts occur annually but are seasonally constrained, reducing the number of exposures occurring at sites. However, for ecosystems where outbreaks occur infrequently (e.g. decadal periods in Kruger National Park, South Africa [24]; sporadic events in Ruaha National Park, Tanzania [49]; re-emergence after 70 years in northwest Siberia [50]), understanding the cause of disease emergence with such a long time-lag between the last recorded case is more difficult to understand. While anthrax can survive for decades in the soil, the concentration of spores is reduced and the risk of an effective contact resulting in anthrax mortality is low. Environmental perturbations may drive outbreaks with time lags that exceed the lifespan we detected for surface soils of reservoirs. Extreme events such as droughts, floods, or permafrost melting may impact host–pathogen contact rates, exposure doses or host susceptibility. Anthropogenic disturbances can also enhance exposure risk, such as when soil scarification brought *B. anthracis* spores from historic burial grounds—protected in deeper soil layers for 45 years—to the surface, causing anthrax cases [51]. Outbreak emergence decades after the last recorded case may be due to a ‘series of unfortunate events’ that result in infections when contact rates and exposure dose are relatively low or improbable (similar to the alignment of conditions promoting disease spillover [52]).

The lethal dose required to kill a host in natural settings varies with individual susceptibility and behaviour [26]. Using host behaviour combined with pathogen concentrations and knowledge of long-term outbreak patterns, we were able to refine our estimation of the lethal dose of *B. anthracis* in free-ranging wildlife. Anthrax persists endemically and from estimates of the frequency at which animals are exposed to different doses of the pathogen, we can infer lethal doses, in this case between 10^7^ and 10^8^ spores that would give a frequency of infection matching the observed dynamics. This information would otherwise be difficult to determine without expensive and impractical animal trials on large, long-lived wildlife species. Our models assumed infection occurs after a single exposure, without considering previous exposures, and that the lethal dose is fixed over the simulation for all individuals. Lethal doses may vary among and within individuals, depending on factors that alter immune function such as age, sex, reproductive status, nutrition or other infections [53]. Herbivores show evidence of exposure to sublethal doses of *B. anthracis* [28,54]; however, whether these sublethal exposures confer immune protection or how the timing of previous exposures alters susceptibility to subsequent exposures in wild populations remains unknown. If previous exposure builds resistance [55,56], this would reduce the *R* by increasing the lethal dose required for mortality. For inhalational anthrax, experimental daily exposure of New-Zealand white rabbits (*Oryctolagus cuniculus*) to low spore concentrations resulted in death when an accumulated dose, lower than required for a single exposure [56], was reach over a three-week study period [57]. Adding multiple exposures would complicate the computation of *R* as grazing at multiple infectious sites may occur before infections takes place, making it harder to track the number of secondary infections occurring from a single infectious site.

By assessing the *R* for *B. anthracis* infectious patches, we show that heterogeneities in hosts, pathogen and environment are highly interconnected, leading to strong temporal variation in disease dynamics. Variation in these interactions may explain outbreak differences observed in different ecosystems, where host–pathogen exposure rates under dry versus wet conditions change due to herbivore foraging behaviour altered by rainfall variability and vegetation dynamics overlaid upon a landscape of variable pathogen risk. Estimating *R* using multiple data sources demonstrated how heterogeneity of the disease system, notably the variability in the transmission potential of infectious sites, altered epidemiological dynamics. Understanding this heterogeneity may be of importance for risk estimates and control efforts in animal husbandry and conservation in anthrax-prone areas. Future work on how the lethal dose may be connected to recurrent sublethal exposures and general animal health may further explain anthrax dynamics in arid areas worldwide.

Recent studies have recognized the importance of linking fine-scale host movement to disease transmission [15,58], where excluding individuals’ movement decisions can then lead to incorrect predictions [14,59]. Simulation models showed that including feedback into wild boar (*Sus scrofa*) movement decisions led to significantly different results about persistence of classical swine fever, which has severe consequences for disease management [60]. For ETPs specifically, infectious patches can be quite small compared to host ranges [61] and correctly characterizing contacts between host and pathogen is crucial to better inform disease transmission risk. Connecting the three sides of the epidemiological triangle, i.e. connecting how environmental changes affect pathogens, hosts and host–pathogen contacts in heterogeneous landscapes may be of high importance to increase accuracy in disease risks predictions [15]. For Hendra virus (*Hendra henipavirus*), spillover from Australian flying foxes, or fruit bats (*Pteropup* spp.) to horses to humans, is associated with habitat loss and food shortages caused by land use change and climate change [62]. These changes shift bat movements and foraging behaviours, increasing spillover events. Similarly, our study emphasizes the importance of the environmental compartment, notably how seasonality affected pathogen reservoir infectivity and host movements and foraging decisions, with implications for disease transmission risk at seasonal and interannual scales.

## Data Availability

The data and codes used in this manuscript are available at https://github.com/ameliedolfi/Reproduction_number, and on Dryad [63]. Supplementary material is available online [64].
